# The Presence and Distribution of TRPM7 in the Canine Mammary Glands

**DOI:** 10.3390/ani10030466

**Published:** 2020-03-11

**Authors:** Sungin Lee, Seulji Lee, Aeri Lee, Hun Ju Sim, Geon A. Kim, Byung-Jae Kang, Wan Hee Kim

**Affiliations:** 1 Department of Veterinary Clinical Sciences, College of Veterinary Medicine and Research Institute for Veterinary Science, Seoul National University, 1 Gwanak-ro, Gwanak-gu, Seoul 08826, Korea; leesungin@snu.ac.kr (S.L.); fw21550@snu.ac.kr (S.L.); gjswn0302@snu.ac.kr (H.J.S.); pshsje03@snu.ac.kr (G.A.K.); bjkang81@snu.ac.kr (B.-J.K.); 2 Seeu Animal Medical Center, 24, Ichon-ro 64 gil, Younsan-gu, Seoul 04427, Korea; aeridvm@gmail.com

**Keywords:** transient receptor potential, TRPM7, canine mammary gland, RT-PCR, Western blot, immunohistochemistry

## Abstract

**Simple Summary:**

Mammary gland tumors are one of the major causes of mortality in dogs. It is, therefore, imperative to clarify the nature of this disease. Transient receptor potential melastatin-subfamily member 7 (TRPM7), a bifunctional ion channel found in human cells, plays a role in normal physiological processes such as cell development, survival, proliferation, differentiation, and migration. However, TRPM7 is also active in several types of cancers in humans, and blocking the expression of this pathway leads to a decrease in the proliferation, migration, and invasion of cancer cells. We have proved the presence of TRPM7 in healthy canine mammary tissues, which could help further studies that aim to establish the relationship between TRPM7 and its physiological and pathophysiological effects in the canine mammary glands and, possibly, tumors.

**Abstract:**

The transient receptor potential melastatin-subfamily member 7 (TRPM7) cation channel is a bifunctional ion channel with intrinsic kinase activity and is ubiquitously expressed in the animal/human body. Accumulated knowledge of TRPM7 suggests that it plays an essential role in normal physiological processes, including the development, survival, proliferation, differentiation, and migration of cells. The aim of this study was to demonstrate the presence and expression patterns of TRPM7 in normal canine mammary glands using reverse transcription-polymerase chain reaction (RT-PCR), Western blotting, and immunohistochemistry. Normal mammary gland tissue samples were obtained from five female beagle dogs. RT-PCR and sequencing of the amplified PCR products demonstrated the presence of TRPM7 mRNA in normal mammary glands, and the presence of TRPM7 protein was confirmed by Western blotting. Immunohistochemical investigations demonstrated the expression of TRPM7 in the apical membrane of acinar and ductal epithelial cells in the canine mammary glands. These results provide the first evidence of the presence and distribution of TRPM7 in the canine mammary gland and could help explain the physiological and pathological roles of TRPM7 in the canine mammary gland; however, additional studies are required to elucidate these roles.

## 1. Introduction

Ion channels are ubiquitous transmembrane proteins that facilitate the selective transport of ions and solutes across the plasma membrane, or between different intracellular compartments [1]. These channels play important roles in essential physiological functions such as muscle and nerve excitation, cell proliferation, sensory transduction, hormonal secretion, and water and sodium balance regulation [2]. It is suggested that one of these ion channels, the transient receptor potential channels (TRP channels), plays an important part in the pathophysiology of various diseases such as cancer by modulating ion-entry driving forces, and the Mg^2+^ and Ca^2+^ transport machinery in the cell membrane associated with cell proliferation and apoptosis [3,4,5,6]. The transient receptor potential (TRP) channels are divided into seven subfamilies based on amino acid sequences, including TRPA (“A” for ankyrin), TRPC (“C” for canonical), TRPM (“M” for melastatin), TRPML (“ML” for mucolipin), TRPN (“N” for mechanoreceptor potential C), TRPP (“P” for polycystic), and TRPV (“V” for vanilloid). Among these, the subfamily TRPM7 is the most extensively studied ion channel.

The TRPM7 channel is distinctive as a bifunctional protein and consists of an enzymatically active kinase domain and an ion transport domain [7]. The expression of the TRPM7 channel has been reported in almost all human cell lines and tissues, including the mammary glands, indicating that TRPM7 may be a key factor responsible for various physiological functions in each organ [6,8,9,10]. This channel has been characterized as a divalent cation-permeable ion channel essential for Mg^2+^ intracellular homeostasis, and it is related to various physiological processes and cellular responses [6,11]. Recently, new knowledge pertaining to TRPM7 has emerged, linking it to cell proliferation, survival, and development, and identifying its role as a pathophysiological modulator in several disease conditions such as ischemic stroke [12], hypertension [13,14], defective ossification [15], and cancers [16,17,18,19]. In particular, these studies are prominent in the field of cancer; in fact, inhibition of TRPM7 expression impairs the proliferation, migration, and invasion of breast cancer cells [9,10]. Based on these results, several researchers have suggested that TRPM7 may act as not only a biomarker for early diagnosis but also a therapeutic target.

To the best of our knowledge, although earlier research performed in both animals and humans has revealed the expression of TRPM7 in various organs, there is no information regarding the relationship between TRPM7 expression and canine mammary glands. The objective of this study was to demonstrate the presence and distribution of TRPM7 in the normal canine mammary gland by using reverse transcription-polymerase chain reaction (RT-PCR), Western blotting, and immunohistochemistry. The present study could lay a foundation for further studies on the role of the TRPM7 channel in both normal physiological and abnormal conditions of the mammary gland, namely tumors. 

## 2. Materials and Methods

### 2.1. Sample Preparation

In the present study, we used five intact female 2-year-old laboratory female beagle dogs, with no previous experience of pregnancy, housed in the Department of Veterinary Surgery, College of Veterinary Medicine at Seoul National University (SNU), to collect normal mammary gland tissues. Only laboratory dogs confirmed to be in the anestrus period, determined using the vaginal smear test, were included in this research. All dogs included in this study were confirmed to be healthy, based on physical examination, complete blood counts, serum biochemistry, and imaging tests such as thoracic and abdominal X-rays, and abdominal ultrasonography. To analyze the presence and distribution of TRPM7, the mammary gland tissue samples were obtained from the five beagle dogs that were euthanized due to reasons (SNU-181214-3) that would not affect the results of this study. Twenty mammary gland samples from each dog were aseptically collected through right unilateral mastectomy immediately after euthanasia and were divided into two portions randomly. Ten mammary glands were promptly frozen in liquid nitrogen, and then stored at −80 °C until needed for RT-PCR and Western blotting. For immunohistochemical investigation, the remaining ten mammary glands were fixed with 10% neutral buffered formalin for 24 h at room temperature, and subsequently embedded in paraffin blocks. All mammary gland samples were reconfirmed to be normal by histopathological examination (hematoxylin and eosin staining). All procedures in this study were approved by the Seoul National University Institutional Animal Care and Use Committees (SNU-190930-8).

### 2.2. Reverse Transcription-PCR

The total RNA was extracted from the mammary gland samples using the Hybrid-R RNA Extraction kit (GeneAll Biotechnology, Seoul, Korea) according to the manufacturer’s instructions, and the extracted total RNA concentration was quantified using an automated microplate spectrophotometer (Epoch, BioTek Instruments Inc., Winooski, VT, USA). cDNA synthesis was performed using the PrimeScript™ First Strand cDNA Synthesis kit (6110A, Takara Bio, Tokyo, Japan) with 1000 ng of extracted total RNA as a template, and each synthesized cDNA template was amplified using conventional PCR with GeneAmp^®^ PCR System 9700 (Applied Biosystems; Thermo Fisher Scientific, Foster City, CA, USA). The total PCR mixtures (20 μL) contained 10 μL of PCR Premix (i-starTaq, iNtRON, Sungnam, Korea), 1 μL of cDNA, 7 μL of DNase/RNase-free distilled water (Ultrapure^TM^, Thermo Fisher Scientific, Waltham, MA, USA), and 1 μL of each gene-specific primer (forward and reverse) (Standard Oligo, Bioneer, Daejeon, Korea). A specific primer for canine TRPM7 was designed using Primer 3 Software [20] with the following criteria: *T*_m _ of  forward and reverse primers at 57.84 and 58.11 °C, respectively, 20 bp long, close to the 3′ end, GC content in the forward primer 55% and reverse primer 50%, and PCR product 373 bp. Its specificity was checked with the National Center for Biotechnology Information (NCBI) Primer-BLAST [21]. As a negative control, sterile DNase/RNase-free distilled water was used to evaluate contamination by other DNA. In addition, to confirm the integrity of the extracted RNA, amplified DNA fragments of the housekeeping gene, glyceraldehyde 3-phosphate dehydrogenase (GAPDH), were used as the positive controls. Primer sequences used in this study are shown in Table 1. The PCR amplifications were conducted under the following cycling conditions: initial denaturation at 94 °C for 5 min, followed by 35 cycles of denaturation at 94 °C for 40 s each, annealing at 60 °C for 40 s, extension at 72 °C for 1 min, and final elongation at 72 °C for 7 min. The PCR amplicons were analyzed by electrophoresis on a 1.5% agarose gel (Agarose LE, iNtRON, Sungnam, Korea). 

The PCR amplicons specific for canine TRPM7 were verified by DNA sequencing. Purification and isolation of the amplified PCR products from 1.5% TAE agarose gels were conducted using the PureLink™ Quick Gel Extraction and Purification Combo kit (K220001; Invitrogen, Carlsbad, CA, USA) according to the manufacturer’s protocols, and subsequently sequenced.

### 2.3. Western Blotting 

The proteins were extracted from canine mammary gland tissue samples by homogenization in radioimmunoprecipitation assay lysis and extraction buffer (Sigma–Aldrich, Saint Louis, MO, USA) according to the manufacturer’s instructions. The protein concentrations were determined with the BCA Protein Assay Kit (Pierce, Rockland, NY, USA). For denaturation, the extracted proteins (20 µg) were mixed with sodium dodecyl sulfate (SDS) loading buffer (GenDEPOT, Barker, TX, USA) and boiled at 100 °C for 5 min before loading. The samples were run on a 10% SDS-polyacrylamide gel and transferred onto Amersham™ Protran™ nitrocellulose blotting membrane (GE Healthcare Bio-Sciences, Pittsburgh, PA, USA). The membranes were blocked with 5% skim milk (BD, Franklin Lakes, NJ, USA) for 1 h at room temperature and probed overnight at 4 °C with the primary goat polyclonal anti-TRPM7 antibody (ab729, 1:500, Abcam, Cambridge, UK) and mouse monoclonal anti-β-actin antibody (sc-47778, 1:5000, SantaCruz Biotechnology, CA, USA) as the loading control. After washing with Tris-buffered saline with Tween 20, the membrane was incubated with secondary antibodies conjugated with horseradish peroxidase (GenDEPOT, Barker, TX, USA). The protein expression was observed by chemiluminescence with ECL detection reagent (Advansta, Manlo Park, CA, USA) and visualized under the ImageQuant LAS 4000 mini biomolecular imager (GE Healthcare Bio-Sciences, Pittsburgh, PA, USA).

### 2.4. Immunohistochemistry

Neutral buffered formalin-fixed (10%) paraffin-embedded canine mammary gland tissues were cut into 4-µm-thick sections. These sections were placed in an incubator at 60 °C, and then dewaxed with xylene and rehydrated through a graded series of ethanol (100%, 100%, 90%, 80%, 70%, and tap water for 3 min each). Dewaxed, rehydrated tissue sections were incubated for 20 min, and antigen retrieval was carried out using the 2100-retriever pressure cooker (PickCell Laboratories, Amsterdam, the Netherlands) in 10 mM citric acid (pH 6.0) buffer. Peroxidase-blocking (3% hydrogen peroxide) solution was applied to tissue sections for 15 min to block endogenous peroxidase activity. Nonspecific primary antibody binding was quenched by immersing the tissue sections in normal horse serum (10% *v*/*v* in phosphate-buffered saline) for 1 h. 

The sections were incubated overnight at 4 °C with goat polyclonal anti-TRPM7 antibody (1:300; ab729, Abcam, Cambridge, MA, USA), and goat IgG polyclonal antibody (ab37373, Abcam, Cambridge, MA, USA) to serve as the negative control. The sections were exposed to the secondary antibody horseradish peroxidase (HRP)-conjugated anti-goat IgG (ImmPRESS™ HRP Anti-Goat IgG Polymer Detection Kit, MP-7405, Vector Laboratories, Burlingame, CA, USA) for 1 h. They were developed with 3,3′-diaminobenzidine (DAB Peroxidase Substrate kit, SK-4100, Vector, CA, USA) for colorimetric visualization of antigen, and the slides were counterstained with Mayer’s hematoxylin. The C57BL/6J mouse brain was used as a positive control.

The immunostained sections were analyzed and photographed using an optical microscope (OlympusBX50, Olympus Optical Co., Tokyo, Japan) connected to a high quality, efficiently cooled, scientific CMOS camera FL-20 (TucsenPhotonics, Gaishan Town, China).

## 3. Results

### 3.1. Reverse Transcription-PCR

**Figure 1:**
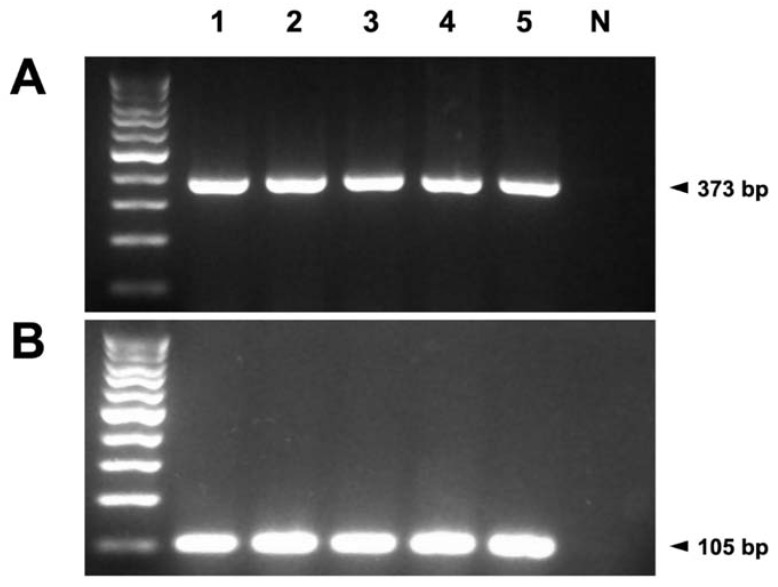
(**A**) mRNA of TRPM7 was detected in five (1–5) canine mammary gland tissues by reverse transcription-polymerase chain reaction (RT-PCR). The amplified products of TRPM7 (373 bp) were observed in all canine mammary gland samples. (**B**) To confirm the integrity of the extracted RNA, the amplified products of glyceraldehyde 3-phosphate dehydrogenase (105 bp) were used as the positive controls. The negative controls (N) are the last lanes on the right.

**Figure 2:**
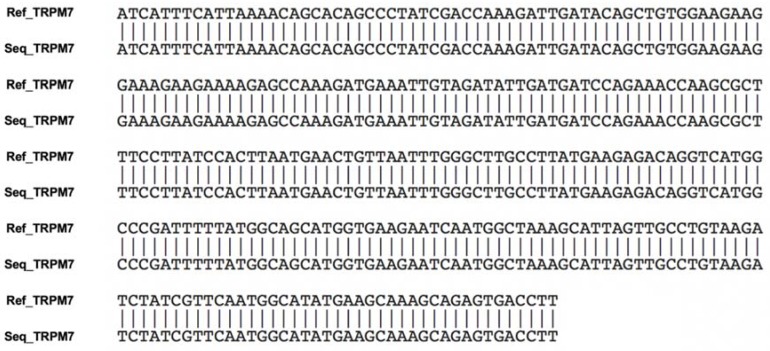
Nucleotide sequences of the amplified PCR products. Ref_TRPM7 indicates the TRPM7 sequences obtained from the National Center for Biotechnology Information sequence database. Seq_TRPM7 indicates the TRPM7 sequences obtained using RT-PCR in the present study. Each nucleotide sequence matched the expected sequences of canine TRPM7.

The presence of mRNA encoding TRPM7 was confirmed in all the normal canine mammary gland tissue samples (Figure 1A). GAPDH, used as the positive control, was amplified simultaneously, whereas the negative control samples were not amplified (Figure 1B). These results confirmed good RNA integrity and proper product amplification of TRPM7 (Figure 2). 

### 3.2. Analysis of Amplified PCR Products 

The sequence of each amplified PCR product was analyzed using the ClustalW multiple sequence alignment program. The sequence of each amplified PCR product matched the published sequences of canine TRPM7 from the National Center for Biotechnology Information (Figure 2).

### 3.3. Western Blotting 

**Figure 3:**
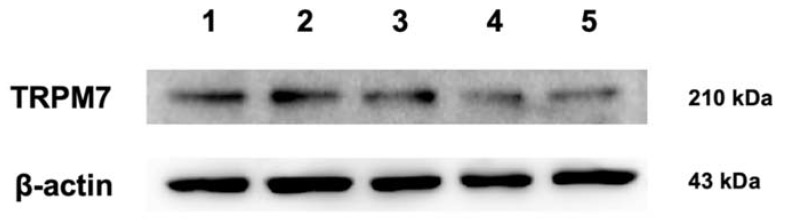
Protein expression of TRPM7 in the five (1–5) canine mammary gland tissues revealed by Western blotting. The immunoreactive bands of approximately 210 kDa were confirmed in all samples. β-Actin (43 kDa) was used as the loading control.

The expression of the TRPM7 protein was confirmed by immunoreactive bands of approximately 210 kDa in all the normal canine mammary gland tissue samples studied (Figure 3). The expression of β-actin was used as the loading control and was also detected simultaneously.

### 3.4. Immunohistochemistry

**Figure 4:**
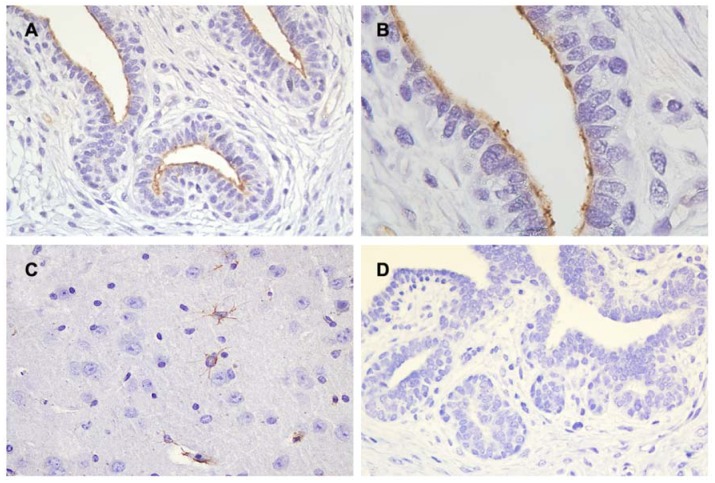
TRPM7 (**A**,**B**) immunohistochemistry in the canine mammary gland. Immuno-positive staining localized in the apical membrane of ductal epithelial cells (**C**) In the positive control, positive immune-reactions were observed in the perinuclear compartments and cytoplasm of the neurons in the mouse brain. (**D**) No specific staining was observed in the negative control. Cell nuclei were stained with Mayer’s hematoxylin. (A, C, D original magnification ×400; B original magnification ×1000).

The immunohistochemical analysis revealed positive immunoreactivity for TRPM7 (Figure 4A,B) in the normal canine mammary gland. Specific immuno-positive staining of TRPM7 was observed on the apical membrane of ductal epithelial cells. In the positive control, specific immune-staining of TRPM7 was confirmed in the perinuclear regions and cytoplasm of the neurons in the mouse brain (Figure 4C) [22]. However, no positive reactions for TRPM7 were observed in the negative control (Figure 4D).

## 4. Discussion

The results of this study confirmed the presence of TRPM7 in normal canine mammary glands at the mRNA and protein levels through RT-PCR and Western blotting, respectively. Previous research on mouse and human cell lines and tissues has reported the expression of TRPM7 transcripts throughout the animal/human body [23,24,25,26]. TRPM7 is most abundantly expressed in the human heart, pituitary glands, bones, and adipose tissues, as observed by quantitative real-time RT-PCR [8]. In addition, the presence and distribution of TRPM7 in normal human breast tissue was also confirmed at the mRNA and protein levels by studies carried out on human breast cancer [9,18]. In our study, the size of the immunoreactive band confirmed by Western blotting was approximately 210 kDa. This result was consistent with that of several previous studies that performed Western blotting using the same antibody for TRPM7 (anti-TRPM7 antibody ab729) [27,28,29]. This is also supported by a review showing that the TRPM7 protein contained six transmembrane segments, each formed of approximately 21 amino acids and composed of 1865 amino acids of molecular weight 210 kDa [30]. Although TRPM7 orthologs have been identified in *Canis lupus familiaris* (dog) [26], this is the first study to prove the presence of TRPM7 in normal canine tissues.

Immunohistochemistry was conducted to validate protein location, and TRPM7 was found to be located in the apical membranes of ductal epithelial cells. This is different from the findings of previous studies on normal human mammary glands and mammary gland tumors, wherein it was mainly located in the perinuclear membrane and cytoplasm [9,18]. TRPM7 is known as the integral membrane-protein and immuno-stained at both punctate membrane and cytoplasm, mainly in the plasma membrane [24]. The intracellular location of TRPM7 varies depending on the kinase activity and phosphorylation state of cells [7]. In a previous study that evaluated the differences in TRPM7 expression among normal pancreas, chronic pancreatitis tissue, pre-malignant tissues, and malignant neoplasms (pancreatic adenocarcinoma, pancreatic adenosquamous carcinoma, solid pseudopapillary neoplasm, and acinar cell carcinoma), the expression level and localization of TRPM7 varied depending on the pathophysiological state of the pancreas [11]. In addition, internalization of normal plasma membrane TRPM8 was observed in the prostate tumor, and internalization and subsequent degradation of cancer cells were correlated with the severity of prostate cancer [31,32]. Based on the conclusions of these studies, it is expected that the localization of TRPM7 in normal canine mammary glands would be a basis for studying the expression pattern of TRPM7 under pathologic conditions including mastitis and mammary gland tumor.

Among the TRPM channels, TRPM7 has been widely studied and found to be the most extensively located channel. In concert with its presence at various locations, TRPM7 is known to perform a variety of physiological functions. TRPM7 is known to govern the survival and anoxic death of various type of cells, such as neurons [33], hepatic cells [34,35], and bone marrow-derived mesenchymal stem cells [36]. In addition, some of these studies demonstrated that these functional roles of TRPM7 were induced by mitogens, oxidative and mechanical stresses, and inflammatory cytokines [35,37]. Therefore, it is suggested that TRPM7 plays an important role in responding, protecting, and maintaining the homeostasis under physiological or external changes and stimuli. Furthermore, it has a role in developing and performing specific organ functions. TRPM7 has a critical role in early cardiogenesis; it also affects the gene expression in the myocardium and subsequently, influences ventricular function, conduction, depolarization, repolarization, and automaticity [38,39]. TRPM7 is also located in the small intestine and colon, as it is required for intestinal pacemaking and peristalsis [40,41]. Other studies have demonstrated that TRPM7 has several specific functions, including synaptic neurotransmissions [42,43] and bone growth [44,45]. To date, however, the physiological function of TRPM7 in the normal mammary gland has not been studied, and therefore, further studies are required.

In human medicine, previous research on TRPM7 has suggested that it acts as a pathophysiological regulator in several diseases including various types of tumors. A change in the level of expression occurred in pancreatic adenocarcinoma and breast cancer [11,18], and the mutation of TRPM7 was identified in colon cancer [46] and ovarian carcinoma [47]. In addition, TRPM7 expression was positively associated with the size, stage, and histological grade in pancreatic adenocarcinoma and breast cancer in humans [11,18,19]. One study showed that the deficiency of TRPM7 in human pancreatic cancer cells resulted in impaired proliferation and arrested the G_0_/G_1_ phases of the cell cycle by regulating Mg^2+^-sensitive suppressors of cytokine signaling 3a pathway [48]. Another study demonstrated that TRPM7 is involved in the proliferation of retinoblastoma cells and in the progression of the G_1_/S cell division cycle by modulating spontaneous activated Ca^2+^-influx pathways [16]. TRPM7 also modulates pathophysiological mechanisms in the mammary gland, either indirectly or directly, including regulation of myosin II-based cellular tension or mitogen-activated protein kinases, as well as being involved in the migration and invasion of breast cancer cells [9,10]. Intracellular calcium regulated by TRPM7 also induces epithelial–mesenchymal transition (EMT), converting epithelial cells to mesenchymal-like cells, crucial for the metastasis of breast cancer [49]. Furthermore, a research using ovarian cancer confirmed TRPM7 silencing reduces the EMT process, which eventually reduces migration, invasion, and wound healing of cancer cells [50]. In addition, the fact that the blockade of TRPM7 or suppression of TRPM7 expression induced the inhibition of cancer cell growth and survival was verified in gastric, breast, and squamous cell carcinoma [51,52,53]. Taken together, these results suggest that an aberrant TRPM7-mediated signaling mechanism is associated with oncogenesis and metastasis and that TRPM7 could have potential as a predictive biomarker and therapeutic target.

Although various disorders related to aberrant expression of TRPM7 have been increasingly researched, their exact pathogenesis and etiology are not fully understood. As in humans, the mammary gland tumor is one of the most life-threatening tumors in dogs. The results of our study and various studies on the physiological and pathological functions of TRPM7 may be key to understanding the physiology and pathology of canine mammary glands. Further studies are needed to investigate the role of TRPM7 in the mammary glands, and to determine its value as a prognostic factor and a therapeutic target. 

## 5. Conclusions

In conclusion, we demonstrated the presence of mRNA and protein of TRPM7 in the canine mammary glands. We also confirmed that TRPM7 was localized in the apical membrane of acinar and ductal epithelial cells. The results prove the presence and distribution of TRPM7 in normal canine tissues and suggest that the canine mammary gland tissue may be affected by TRPM7. These findings could increase our understanding of the associations between TRPM7 and normal physiological roles of the mammary gland tissue as well as the pathophysiological mechanisms of canine mammary gland diseases such as inflammation and tumor characterized by TRPM7 system dysfunction.

## Figures and Tables

**Table 1:** Specific oligonucleotide primer sequences used for reverse transcription-polymerase chain reaction with amplicon sizes in this study.

Target Gene	Accession Number	Primer Sequence (5′ to 3′)		Amplicon Size (bp)
		Forward	Reverse	
GAPDH	NM_001003142.2	CATTGCCCTCAATGACCACT	TCCTTGGAGGCCATGTGGAC	105
TRPM7	XM_022413007.1	CTGGCCGAAATACCTCTAGC	AGGTCACTCTGCTTTGCTTC	373

GAPDH, glyceraldehyde-3-phosphate dehydrogenase; TRPM7, transient receptor potential cation channel, subfamily M, member 7.
